# 1608. Real-World Effectiveness of Long-Acting Cabotegravir + Rilpivirine in Virologically Suppressed Treatment-Experienced Individuals: Two Years of Data from the OPERA^®^ Cohort

**DOI:** 10.1093/ofid/ofad500.1443

**Published:** 2023-11-27

**Authors:** Michael Sension, Ricky K Hsu, Jennifer S Fusco, Laurence Brunet, Quateka Cochran, Gayathri Sridhar, Vani Vannappagari, Jean A van Wyk, Michael B Wohlfeiler, Gregory P Fusco

**Affiliations:** CAN Community Health, Fort Lauderdale, Florida; AIDS Healthcare Foundation\ NYU School of Medicine, New York, New York; Epividian, Inc., Durham, North Carolina; Epividian, Inc., Durham, North Carolina; Aids Healthcare Foundation, Miami Beach, Florida; ViiV Healthcare, Fairfax, Virginia; ViiV Healthcare, Fairfax, Virginia; ViiV Healthcare, Brentford, UK, Brentford, England, United Kingdom; AIDS Healthcare Foundation, Miami Beach, Florida; Epividian, Inc., Durham, North Carolina

## Abstract

**Background:**

Cabotegravir + rilpivirine (CAB+RPV) injections, the first complete long-acting (LA) antiretroviral therapy (ART) regimen, was approved by the FDA in January 2021 for ART-experienced people with HIV (PWH) who are virologically suppressed (viral load [VL] < 50 copies/mL). We assessed the virologic effectiveness of CAB+RPV LA among ART-experienced individuals with VL < 50 copies/mL at initiation in the first 2 years of use in the OPERA® Cohort, stratified by body mass index (BMI).

**Methods:**

All ART-experienced adults with VL< 50 copies/mL at initiation who received ≥ 1 CAB+RPV LA injection for the first time between 21Jan2021 and 28Feb2023 were followed until 25Mar2023. Individuals on either monthly or every 2-month injection schedules were included. Discontinuation was defined as a regimen switch or 2 consecutive missed injections. VLs were monitored from first injection until CAB+RPV discontinuation, death, or study end. Confirmed virologic failure (CVF) was defined as 2 consecutive VLs ≥ 200 copies/mL or 1 VL ≥ 200 copies/mL followed by discontinuation. Results were stratified by BMI at first injection (< 30 vs. ≥ 30 kg/m^2^).

**Results:**

All 1843 PWH who received CAB+RPV injections were ART-experienced at start and 1578 (86%) had VL < 50 copies/mL at initiation. Of the 1578 suppressed, 267 (17%) were women, 654 (41%) were Black, 451 (29%) were Hispanic, and 469 (30%) had a BMI of ≥ 30; median age was 40 (IQR: 32, 53) years (Table 1). Among those with CAB+RPV dose available, 1297 (84%) remained on CAB+RPV LA over a median follow-up of 7.4 (IQR: 3.9, 10.9) months at study end. Among 1323 with VLs after first injection, the last VL measured was < 200 copies/mL in 99% (n=1305) and < 50 copies/mL in 94% (n=1237) (Table 2); all follow-up VLs were < 200 copies/mL in 96% (n=1272), and < 50 copies/mL in 84% (n=1115). Regardless of BMI, virologic suppression was maintained by ≥ 98% of individuals and only 1% experienced CVF over follow-up (Table 2).

**Figure:**
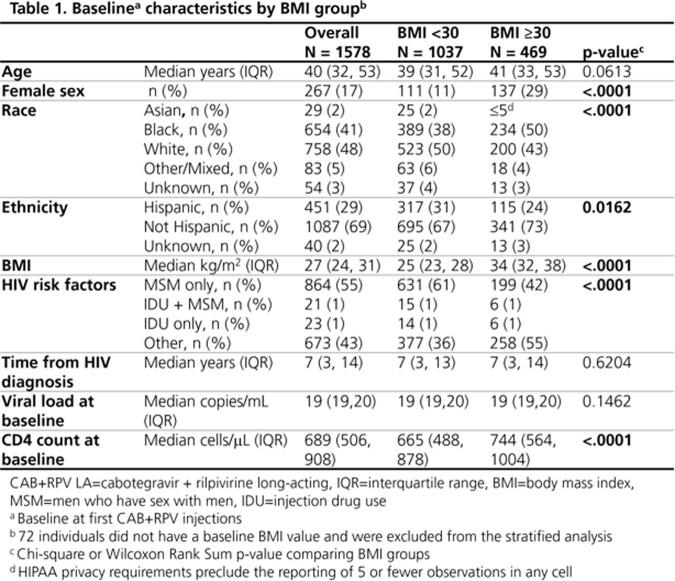

**Figure:**
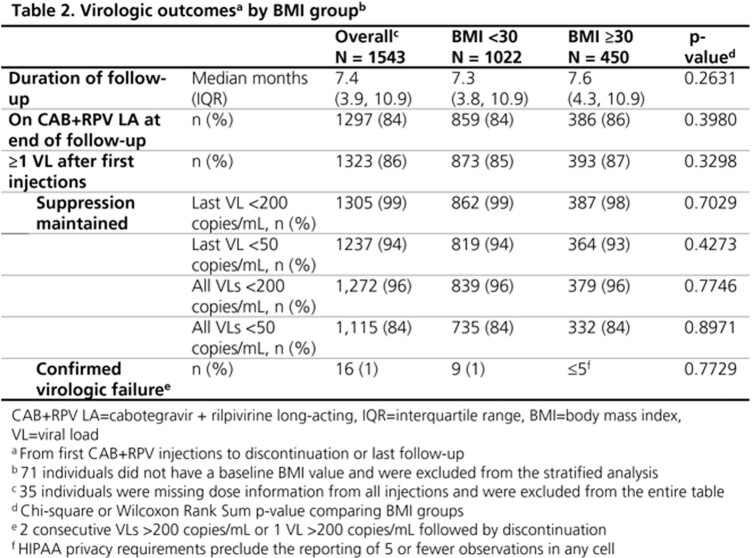

**Conclusion:**

In this real-world cohort of PWH in the United States who received CAB+RPV LA injections, observations from the first 2 years suggest that this regimen is effective among individuals virologically suppressed at initiation. Furthermore, the regimen is consistently effective among those with high BMI.

**Disclosures:**

**Michael Sension, MD**, Gilead: Advisor/Consultant|Gilead: Honoraria|Viiv: Advisor/Consultant|Viiv: Grant/Research Support|Viiv: Honoraria **Ricky K. Hsu, MD**, Gilead Sciences: Advisor/Consultant|Gilead Sciences: Grant/Research Support|Gilead Sciences: Honoraria|Janssen: Advisor/Consultant|Janssen: Grant/Research Support|Janssen: Honoraria|Merck: Advisor/Consultant|Merck: Honoraria|ViiV Healthcare: Advisor/Consultant|ViiV Healthcare: Honoraria **Jennifer S. Fusco, BS**, Epividian, Inc.: Salary|Epividian, Inc.: Ownership Interest|Epividian, Inc.: Stocks/Bonds **Laurence Brunet, PhD**, Epividian, Inc.: Salary|Epividian, Inc.: Stocks/Bonds **Gayathri Sridhar, MBBS, MPH, PhD**, GlaxoSmithKline: Stocks/Bonds|ViiV Healthcare: Full Time Employee **Vani Vannappagari, MBBS, MPH, PhD**, GlaxoSmithKline: Stocks/Bonds|ViiV Healthcare: Employee **Jean A. van Wyk, MBChB, MFPM**, ViiV Healthcare Ltd: Stocks/Bonds **Michael B. Wohlfeiler, JD, MD, AAHIVS**, ViiV Healthcare: Serves as a PI on clinical trials, but does not receive personal compensation for this work (it goes directly to AIDS Healthcare Foundation) **Gregory P. Fusco, MD, MPH**, Epividian, Inc.: Board Member|Epividian, Inc.: Ownership Interest|Epividian, Inc.: Stocks/Bonds

